# State-of-the-Art Molecular Oncology in UK

**DOI:** 10.3390/ijms24119336

**Published:** 2023-05-26

**Authors:** Saleh Sandoughdaran, Christos Mikropoulos, Stergios Boussios

**Affiliations:** 1 St Luke’s Cancer Centre, Royal Surrey Hospital, Guildford GU1 1EB, UK; christos.mikropoulos@nhs.net; 2 Department of Medical Oncology, Medway NHS Foundation Trust, Windmill Road, Gillingham ME7 5NY, UK; stergios.boussios@nhs.net; 3 Faculty of Life Sciences & Medicine, School of Cancer & Pharmaceutical Sciences, King’s College London, London SE1 9RT, UK; 4 Kent Medway Medical School, University of Kent, Canterbury CT2 7LX, UK; 5 AELIA Organization, 9th Km Thessaloniki-Thermi, 57001 Thessaloniki, Greece

Molecular oncology is a rapidly evolving field that focuses on the genetic and molecular basis of cancer. Recent advances in technology and our understanding of molecular pathways have led to significant progress in the diagnosis, treatment, and prevention of cancer. For instance, next-generation sequencing techniques have allowed for the identification of genetic mutations and alterations in cancer cells, leading to the development of targeted therapies. Immunotherapy, which harnesses the body’s immune system to fight cancer, has also shown promising results in treating various types of cancer. Additionally, the discovery of biomarkers has enabled the development of personalized cancer therapies that are tailored to an individual’s unique genetic makeup. Overall, the advances in molecular oncology are transforming the way cancer is diagnosed and treated, providing hope for better outcomes for cancer patients in the future.

This Special Issue of the *International Journal of Molecular Sciences*, entitled “State-of-the-Art Molecular Oncology in the UK”, includes a total of seven contributions, comprising five original articles and two reviews, which provide new information about novel mechanistic insights into cancer molecular pathways.

The study conducted by Pape et al. [1] aimed to investigate the effect of biophysical parameters, specifically oxygen concentration and matrix stiffness, on the expression of epithelial-to-mesenchymal transition (EMT) markers and cancer invasion in HT-29 cells. They found that physiological hypoxia was sufficient to trigger the expression of EMT markers in HT-29 cells in 2D by day 7. In 3D, HT-29 cells invaded more extensively in a stiff matrix environment with corresponding increases in the invasive genes MMP2 and RAE1. The study also found that biophysical parameters can induce phenotypic and genotypic changes in cancer cells, which may have implications for cancer progression. However, it should be noted that this was a preliminary study, and further research will be needed to fully understand the implications of these findings for cancer therapy development.

Anpalakhan et al. [2] investigated the effects of GCSF primary prophylaxis on survival outcomes and toxicity in patients with advanced non-small cell lung cancer on first-line chemoimmunotherapy. The study was conducted as a sub-analysis of the Spinnaker study and involved multiple participating sites. The results showed that GCSF prophylaxis did not improve overall survival or progression-free survival; however, it increased the rate of Grade 1–2 adverse effects, including immunotherapy-related toxicities. The study provides valuable insights into the risks of GCSF prophylaxis in NSCLC patients receiving chemoimmunotherapy, and can inform clinical practice to improve patient care for these group of patients.

The importance of the Ras-related nuclear protein (Ran) in cellular signalling and malignant phenotyping is the topic of the review performed by El-Tanani et al. [3]. The Ran cycle is made up of the two states of Ran, Ran GTP and Ran GDP, which may be a member of the Ras superfamily of proteins. Both the conversion of GDP to GTP by the regulator of the chromosome condensation 1 (RCC1) guanine nucleotide exchange factor and the activation of Ran’s intrinsic GTPase activity through association with Ran guanine-activating proteins (Ran GAP) and Ran-binding proteins, which results in the hydrolysis of GTP, maintain the balance between these two states. Ran controls a number of critical functions, including nuclear transport, spindle assembly during mitosis, and microtubule organisation, through the course of this cycle. The review addresses many Ran-related pathways that may be investigated for cancer therapy. However, further research is required to fully understand the mechanisms involved and to develop effective therapies targeting Ran GTPase in cancer treatment.

Yang et al. [4] conducted a study to investigate the potential role of the activated leukocyte cell adhesion molecule (ALCAM) in the peritoneal metastasis of gastrointestinal cancers. Specifically, they aimed to determine whether ALCAM expression in gastrointestinal cancers, including gastric and pancreatic cancers, has a predictive value in the development of peritoneal metastasis. They also sought to elucidate the mechanisms underlying ALCAM-mediated tumour–mesothelial interactions and explore the therapeutic implications of targeting ALCAMs for cancer treatment. According to their findings, ALCAM proteins operate as “seed” receptors on cancer cells and “soil” receptors on peritoneal mesothelial cells via homotypical protein–protein interactions. Cancer cells settle across the peritoneum as a result of ALCAM-mediated tumour–mesothelial contact; this mechanism is probably dependent on SRC kinase signalling. The study also proposed that targeting ALCAMs represents a novel therapeutic opportunity in both preventing and treating peritoneal metastases.

The evaluation of EPLIN in colorectal cancer and its mechanism of action was the innovative subject of the study of Zeng et al. [5]. The study found that EPLIN is downregulated in colorectal cancer tissues compared to normal tissues and that this reduced expression is associated with poor clinical outcomes. In vitro cellular function assays showed that EPLIN inhibited cellular growth, adhesion, migration, and invasion. These findings also suggest that Her2 and HSP60 are EPLIN’s interacting partners, although no direct protein–protein interaction was observed.

The review by Eltarhoni et al. [6] provides an overview of the development and use of monoclonal therapeutic antibodies to target multiple cancer pathways. The authors thoroughly discuss the molecular mechanisms of carcinogenesis and how this understanding has led to the development of recombinant protein therapeutics, which now constitute a large proportion of yearly approved medicines. The paper also highlights some of the most promising therapeutic antibodies currently being used in cancer treatment in the UK, as well as potential side effects and challenges facing their development and implementation. Overall, their review provides valuable insights into the latest developments in cancer treatment using therapeutic antibodies.

Mps1 kinase is a protein kinase that plays a crucial role in the regulation of cell division and chromosome segregation during mitosis. It is overexpressed in many types of cancer and is therefore considered a potential target for the development of new cancer therapeutics. Pugh et al. [7] used a computational biology approach to investigate the dynamics of Mps1 kinase interactions with small-sized molecules of the isoflavone class. The authors identified new potential Mps1 kinase inhibitors with predicted favourable drug-like features, which could lead to the development of more effective therapeutics for the treatment of cancer and other malignancies associated with Mps1 overexpression, defects in chromosome segregation, cell division, and cell proliferation.

Molecular oncology is a vast and constantly evolving field of research. While many areas have been extensively studied, there are still several aspects of cancer biology that remain poorly understood, including non-coding RNAs, epigenetic modifications, the tumour microenvironment, and single-cell genomics. Although papers in this Special Issue address some of these areas, there is still much to be learned about the complex biology of this disease.
